# The New Theory and Prophylaxis of Puerperal Eclampsia
1Read at a meeting of the Bath and Bristol branch of the British Medical Association, March 28th, 1906, at Bristol.


**Published:** 1906-06

**Authors:** Eliza L. Walker Dunbar


### THE NEW THEORY AND PROPHYLAXIS OF PUERPERAL ECLAMPSIA.[1]

BY

Eliza L. Walker Dunbar, M.D.

---

Last July I saw in the country a severe case of eclampsia, and a week after, the sister of this patient wrote to me, saying that her own hands and feet were swollen—that is to say, she herself was displaying the same symptoms that had preceded the eclampsia in the first case in which no prophylactic measures had been taken, for the lady had not sought advice. Both sisters were pregnant for the first time, and were 38 and 39 years of age respectively. The loss of the infant—the pregnancy was in the seventh month, and the fœtus was dead before the necessary accouchement *forcé* could be begun—was a great grief to the mother, and I was very anxious to prevent a second catastrophe in the family. I must not omit to mention that my own patient a few days before the news of her sister's danger reached her had experienced a great shock in the tragic and sudden death of an intimate friend. She had thus suffered a double shock.

A specimen of urine sent by post showed that albuminuria had not set in. I knew it might appear later, and that fatal cases had occurred in which no albuminuria had existed or had not developed until within fourteen days of parturition.

Having signally failed to prevent eclampsia some years ago

[1] Read at a meeting of the Bath and Bristol branch of the British Medical Association, March 28th, 1906, at Bristol.

by treating kidney symptoms—that is with diaphoretics, hot air and milk diet, a treatment most conscientiously carried out for many weeks—I considered what other means were available to prevent the dreaded outcome, and I came to the conclusion that the swelling which precedes eclampsia is due to the heaping up of semi-solid matter in the various tissues, and might be dissipated by thyroid gland substance. I came independently on this idea, but I have since learnt that much the same thought had occurred previously to at least one other doctor—a Dr. Nicholson—but I do not know any details of his experiences, and I understand that the theory requires further proof and corroboration before it can be generally accepted.

From my first thought that the problem to be solved was that of eliminating semi-solid matter, followed as a logical sequence that if thyroid gland substance did eliminate the deleterious matter, the presence of that matter was due to insufficient activity of the thyroid—was in fact myxœdema. It remained to explain why pregnancy should affect the activity of the said gland.

I would not carry out my proposed treatment without personal observation, and it was not until September that I could begin. Meantime, about the middle of August, at the end of the sixth month of pregnancy, the lady's husband awoke one night to find her in a state of convulsion, unconscious, breathing stertorously. The attack subsided, and recurred twice in half an hour—three attacks in all. The patient had been more than usually active during the preceding day and had taken a rather heavy supper. Competent observation showed that the urine was free from albumen and was abundant, nearly a quart a day.

When I saw the patient in September—I had seen her in June and also earlier—she was swollen all over, but more markedly in face, hands and feet. The wedding-ring, which had been so loose that it dropped off sometimes, had to be left off in July on account of swelling of the fingers. Patient complained of headache, inability to read with understanding and pleasure, and felt special discomfort from the numbness and weight of the hands. The pulse was 84 and tense. There was no pitting of the skin on pressure anywhere. The daily quantity of urine was a quart and over. A tabloid of $1\frac{1}{2}$ grains thyroidin (B. W. & Co.) taken twice a day was well borne, and a very slight improvement set in. It became more visible when four tabloids were taken daily, and six tabloids per day effected an alteration for the better that could not be overlooked. Patient was able to read again with profit and delight; she felt more contented; the fingers looked more shapely, the pulse became soft. No deleterious effect on the fœtus was observable.

In October patient had an attack of giddiness—one of the well-known prodromata of eclampsia—the head felt oppressed and dull; but after a very few days the attack passed off, and she declared herself quite well again. As anæmia is suggested as a cause of eclampsia, I determined the hæmoglobin by the Fleischl hæmometer, and found it to be $100^{\circ}$.

As any extra exertion always increased the swelling, care was taken to lessen very much the output of work during the last ten weeks of pregnancy. Three weeks before parturition albumen appeared in the urine quite distinctly, but not to any great extent. Its appearance was a further proof of the presence of the danger of eclampsia. Parturition took place on November 11th quite normally, except that the patient swelled very much during labour, which lasted eight hours. The infant weighed $5\frac{1}{2}$ lb. and was very lively. The milk secretion was particularly abundant. Thyroidin was continued for about a week, and then the urine was free from albumen. Thyroidin produced a much greater effect in reducing swelling after than before the birth. Parturition did not entirely remove the cause of the swelling. The hands are still a little swollen, although rings can be worn. I have begged the patient to let me know when her fingers become thin again, and[1] I conjecture that will be when the catamenia are re-established. In that conjecture lies the suggestion I have to make as to the root cause and additional measures of prevention of puerperal eclampsia.

As long ago as 1870 some interdependence of the thyroid gland and the genital system was recognised. Thus a boy who

[1] This conjecture has proved correct.

came to the Polyclinic with goitre was told it might disappear in two or three years when he grew up, an expectation that was realised; for meeting the lad two or three years later I ascertained that his goitre had disappeared, and by no other means than Nature's development. We were also instructed that disease of the thyroid gland was frequently associated with amenorrhœa. Chlorosis, that malady of development in girls—I read there is an analogous malady in boys—is accompanied by a general swelling, which I hold to be due to defective action of the thyroid gland. The swelling is unlike that of hydræmia, in that it does not pit on pressure; also hydræmia represents a much graver illness than chlorosis is.

During the climacteric change women suffer from some amount of general swelling, which is also unlike that of hydræmia, and is independent of anæmia or kidney disease. I take the cause to be a disturbance of the thyroid gland activity determined by involution of the ovaries. The disturbance subsides generally without special treatment, but sometimes requires temporary assistance.

Here is a case of mine which illustrates the interdependence of ovarian function and thyroid gland activity. The patient was a young lady, aged 24 years, who had had irregular and very scanty catamenia for about a year in spite of administration all that time of iron tonics in abundant quantity. She had evidently myxœdema. Thyroid gland effected a marked change in her appearance. Ovarian substance was also administered, and the catamenia reappeared more punctually and were more natural in appearance. When the menstrual function was fully restored the myxœdema had disappeared.

With such-like considerations I have come to the opinion that the thyroid gland is indeed dependent somehow on the healthy functioning of the genital organs. In women the thyroid requires the contribution of the ovarian secretion to carry on its work. In pregnancy if any function is in abeyance it is that of the ovaries. Does the ovarian secretion perchance include a catalyst that acts with thyroid secretion on waste tissue? With the new knowledge of catalysis who shall say what substance may or may not act as a solvent?

There are persons whose thyroid gland output is naturally below average. They look rather stouter than most persons, but seem quite healthy. In their case the effectiveness of the thyroid is more easily hindered, or it may be that when the demands of the system are increased by pregnancy and the assistance of the ovarian secretion suspended the slight defect of the gland becomes apparent.

Administration of the thyroid gland will reduce the swelling and relieve the obvious pressure on the brain and clogging of other organs. It must be remembered that myxœdema manifests itself not only in the skin, but in the mucous membrane, and presumably in all the organs of the body, including the kidneys. A point to be remembered by critics of the theories I have propounded is that myxœdema seems to have a varied preference for the organ or organs in which it shall act most deleteriously. Students of myxœdema will readily agree that its effects are very variously distributed. Therefore it is not always the brain that is most oppressed, even in pregnancy; and when the brain is not specially affected it may be that eclampsia does not follow when parturition suddenly heaps up deleterious material, but the brain being in that case comparatively free is able to bear the strain.

There is a consensus of opinion that venesection in eclampsia affords relief for a time; that which abstraction of blood effects, whether by venesection or cutting the cervix in the interest of accouchement *forcé*, may be induced beforehand by administration of thyroid gland. That will also relieve the kidneys from the deleterious effect of myxœdematous matter.

As a further cure or preventive of myxœdema in pregnancy, it seems to me that administration of ovarian substance should be tried. I should not give it until after the third month in case of its producing abortion, for ovarian substance is an emmenagogue. The dose should be small and given with caution. It should be tried in conjunction with thyroid gland until it appears that the myxœdema is more than merely kept in abeyance.

The output of work in pregnancy associated with myxœdema should be greatly restricted, for the demand in the activity of the thyroid gland is greater the greater the amount of expended energy. The name puerperal eclampsia, signifying as it does something as unexpected as lightning, can no longer excuse the omission of a prophylactic treatment which has already been effective. The differential diagnosis of myxœdema and nephritis will not be difficult. In myxœdema the quantity of urine is not diminished; even when the kidney is assisting to carry off myxœdematous matter the quantity of urine is not much lessened. The fact of pregnancy being the exciting cause points to the right diagnosis.

---

